# Role of Plant Growth Regulators in Adventitious *Populus Tremula* Root Development In Vitro

**DOI:** 10.3390/plants14152427

**Published:** 2025-08-05

**Authors:** Miglė Vaičiukynė, Jonas Žiauka, Valentinas Černiauskas, Iveta Varnagirytė-Kabašinskienė

**Affiliations:** 1 Institute of Forestry, Lithuanian Research Centre for Agriculture and Forestry, Liepų Str. 1, Kaunas District, LT-53101 Girionys, Lithuania; migle.vaiciukyne@lammc.lt (M.V.);; 2 Department of Biology, Faculty of Natural Sciences, Vytautas Magnus University, Universiteto Str. 10, Kaunas District, LT-46265 Akademija, Lithuania

**Keywords:** aspen, plant tissue culture, phytohormones, shoot-derived roots, lateral roots

## Abstract

Eurasian aspen (*Populus tremula* L.) is a tree species with recognised ecological and economic importance for both natural and plantation forests. For the fast cloning of selected aspen genotypes, the method of plant propagation through in vitro culture (micropropagation) is often recommended. The efficiency of this method is related to the use of shoot-inducing chemical growth regulators, among which cytokinins, a type of plant hormone, dominate. Although cytokinins can inhibit rooting, this effect is avoided by using cytokinin-free media. This study sought to identify concentrations and combinations of growth regulators that would stimulate one type of *P. tremula* organogenesis (either shoot or root formation) without inhibiting the other. The investigated growth regulators included cytokinin 6-benzylaminopurine (BAP), auxin transport inhibitor 2,3,5-triiodobenzoic acid (TIBA), auxins indole-3-acetic acid (IAA) and indole-3-butyric acid (IBA), gibberellin biosynthesis inhibitor paclobutrazol (PBZ), and a gibberellin mixture (GA_4_/_7_). Both BAP and TIBA increased shoot number per *P. tremula* explant and decreased the number of adventitious roots, but TIBA, in contrast to BAP, did not inhibit lateral root formation. However, for the maintenance of both adventitious shoot and root formation above the control level, the combination of PBZ and GA_4/7_ was shown to be especially promising.

## 1. Introduction

Eurasian aspen (*Populus tremula* L.) is described as a tree species of immense ecological importance for the forests of central Europe [1]. Moreover, the recent achievements in investigating the *P. tremula* genome [2,3] have established this species as a suitable model for fundamental studies in tree biology. *P. tremula* is also investigated concerning the possibility of using its wood to produce biofuel [4]. The latter study demonstrated that there is a high genetically determined variation in natural *P. tremula* populations, considering the properties of wood that make it suitable for biorefining. In this context, it is crucial to develop *P. tremula* breeding strategies and, simultaneously, to develop methods for the large-scale propagation of selected *Populus* genotypes.

Micropropagation is often employed as a convenient in vitro method for the vegetative propagation of *Populus* species and hybrids. Besides having the potential to produce high yields of cloned plants from selected genotypes, this method also enables scientists to investigate genotype responses to specific environmental factors, such as light, under controlled conditions [5]. Moreover, in vitro rooted *Populus* explants are a suitable plant material for studying the process of naive root colonisation with bacteria and fungi [6,7].

The conventional methods of plant micropropagation typically involve the use of phytohormones, including cytokinins and auxins, with a higher concentration of cytokinin aimed at promoting shoot regeneration [8]. This process is regulated by cytokinin signalling in *Populus* species [9]. However, cytokinins were shown to block root growth in the model plant *Arabidopsis* [10] and to inhibit lateral root formation in maize [11], as well as to repress adventitious root formation in *Populus* cuttings via misregulation of auxin transport and vascular development pathways [12]. Thus, considering the use of cytokinin for *Populus* micropropagation, its potential to inhibit lateral root formation must also be addressed, especially because lateral roots are essential for the establishment of mycorrhizal symbiosis after a plantlet is transferred to the soil [6]. Although cytokinins are known to potentially inhibit root formation, this effect can be effectively mitigated in *P. tremula* by transferring regenerated shoots to a cytokinin-free rooting medium, resulting in successful root development and plant acclimatisation [13,14,15].

In contrast to cytokinins, auxins are known for their positive role in inducing and promoting root formation [16]. However, it was demonstrated in tissue culture of leaf explants that auxin, even at relatively high concentrations, failed to induce root formation in the presence of cytokinin concentrations that promote shoot development [17]. Besides the use of various natural and synthetic auxins, other auxin-related chemicals are also applied in vitro. For example, auxin prodrugs (chemicals that are inactive in their native form but convert into active auxins within a plant) were shown to promote rooting in *Populus* in vitro cultures [18]. Another group of chemicals may specifically target auxin transport through plant cells and inhibit this process. These chemicals can be used in fundamental studies of tree biology, particularly for investigating the role of auxin in the radial growth of tree stems, which is associated with the formation of water-conducting vessels [19]. Interestingly, the normal formation of vascular tissues in *Populus* stems requires the action of both auxins and cytokinins [20]; however, an in vitro study has shown that exogenously applied cytokinin may even have an inhibitory effect on the broader regulatory activity of auxin [21]. Meanwhile, the application of the auxin transport inhibitor triiodobenzoic acid (TIBA) to the in vitro culture of Arabidopsis seedlings resulted in the induction of new shoot formation [8], suggesting that this growth regulator may serve as an alternative to exogenous cytokinins. Although the interference of TIBA with auxin transport may decrease root formation, some authors [22,23] suggested that disturbed auxin transport may also result in local accumulations of auxin with a potential benefit for lateral root formation. In this context, the present study investigated the effects of TIBA on *P. tremula* shoot explants and how the simultaneous application of auxins alters these effects.

Another pair of plant growth regulators, whose effects on *P. tremula* were investigated in this research, was made of paclobutrazol (PBZ) and gibberellin GA_4/7_. In previous studies on the effects of gibberellins on *Populus* and other plants, gibberellins have been shown to promote plant branching and elongation of axillary buds, acting in coordination with other phytohormones, such as auxins [24], cytokinins [25], and abscisic acid [26]. In both *Populus* and *Arabidopsis*, gibberellins have also been shown to inhibit adventitious rooting, causing this effect through their negative impact on auxin transport [27]. Meanwhile, gibberellin synthesis inhibitor paclobutrazol (PBZ) was shown to affect plants oppositely, as it inhibited bud elongation in *Petunia* [25] and promoted root formation in *Phoebe bournei* (Hemsl.), a difficult-to-root tree species [28]. Thus, considering this pair of growth regulators, the present study investigated whether PBZ had a root-formation-promoting effect on *P. tremula* shoot explants and how the simultaneous application of gibberellin altered this effect.

The knowledge obtained from this study can be applied in practice to address the actual problems of aspen propagation and cultivation. In particular, the efforts to establish short-rotation plantations of aspen through micropropagation require investigation into how the root system development in these trees might be enhanced, making them more adapted to survive ex vitro. Successful propagation systems can be effectively applied to establish valuable plantations of forest trees.

This study aims to investigate how different concentrations and combinations of plant growth regulators affect adventitious root development in *P. tremula* in vitro, with a particular focus on whether the effect of one regulator modifies or interferes with that of another. Although shoot formation was also recorded, the primary objective was to understand synergistic or antagonistic interactions between PGRs in the context of adventitious root development.

## 2. Results

### 2.1. The Effect of Inhibition of Auxin Transport on Root Formation

**Figure 1:**
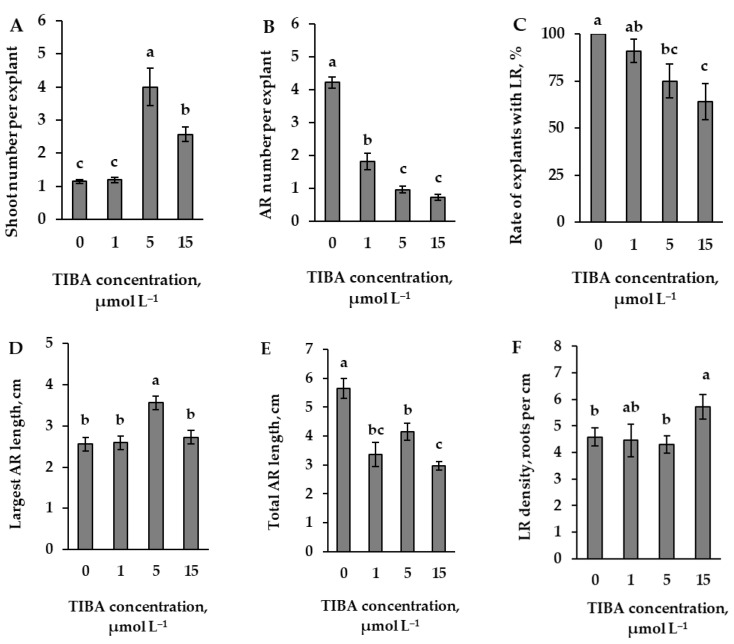
Number of shoots (**A**) and adventitious roots (AR) (**B**) per explant (means ± SE), the rate of explants with lateral roots (LR) (**C**) and main (**D**) and total (**E**) adventitious root lengths (means ± SE) of explants, as affected by the presence of different TIBA concentrations (0, 1, 5 and 15 µmol L^−1^) in the nutrient medium. (**A**–**C**) data are from the total number of explants, and (**D**–**F**) are from the number when the part of the explants with lateral roots exceeds ½ the total number. Lateral root density (LRD) (**F**) was calculated as the number of lateral roots per centimetre of cumulative adventitious root length (LRD = N/L). Significantly different means of samples grown under different nutrient media conditions are labelled with different letters (*p* < 0.05).

In particular, the transport mechanism of auxin was investigated. The results of the determination of the optimal concentration of 2,3,5-triiodobenzoic acid (TIBA), an inhibitor of auxin transport, showed that the lowest 1 μmol L^−1^ concentration resulted in a statistically significant decrease in the number of adventitious roots (Figure 1B) in comparison with data from the control explants. The 1 μmol L^−1^ TIBA concentration did not have a significant effect on the number of shoots. Although the use of higher TIBA concentrations significantly increased the number of shoots, interestingly, the 5 μmol L^−1^ concentration caused a greater increase in shoots than the 15 μmol L^−1^ (Figure 1A). In contrast, the 1 μmol L^−1^ concentration reduced the number of adventitious roots compared to the control explants (Figure 1B). The results of the data from the whole sample of explants indicate that TIBA reduced the rate of explants with lateral roots. In this case, when 1 μmol L^−1^ had no significant effect, when compared with data from control explants, the concentrations of 5 and 15 μmol L^−1^ significantly reduced the rate of explants with lateral roots from 75 or 64%. In the following results for TIBA, the data on the length of the adventitious roots and the density of the lateral roots are analysed only for samples of explants with lateral roots. Therefore, these data were analysed using concentrations of TIBA at 1, 5, and 15 μmol L^−1^ using a methodology based on the rate of the sample with lateral roots forming more than half of the total sample (Figure 1D–F). Only the 5 μmol L^−1^ concentration of TIBA caused a significant increase in the length of the main adventitious root. The effect contrasted with the overall decrease in total root length observed at all concentrations when compared to the control explants (Figure 1D,E). All concentrations of TIBA reduced the number and total length of the adventitious roots; however, with regard to the density of the lateral roots, even when the lower concentrations did not have a significant effect, the 15 μmol L^−1^ concentration increased their density (Figure 1F).

**Figure 2:**
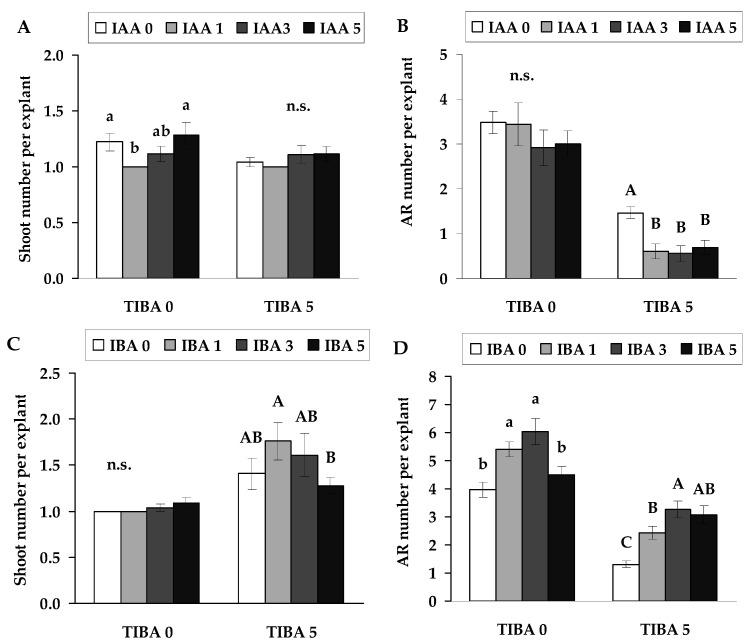
Shoots (**A**,**C**) and adventitious roots (AR) (**B**,**D**) per explant (means ± SE) of explants, as affected by the presence of IAA (**A**,**B**) and IBA (**B**,**D**) at the concentrations of 0, 1, 3 and 5 µmol L^−1^ combination without and with TIBA (5 µmol L^−1^) in the nutrient medium. Significantly different means of samples grown under different nutrient media conditions are labelled with different letters (*p* < 0.05), n.s. indicates not significant (*p* > 0.05).

Based on the effect of TIBA on shoot and root parameters, the optimal concentration of 5 µmol L^−1^ was selected to evaluate TIBA activity in the presence of exogenously applied IAA. The results showed that, despite the presence of higher concentrations of IAA, TIBA retained its inhibitory effect on root formation (Figure 2B). Thus, when applied together, TIBA’s negative effect on root development was not mitigated by IAA, contrary to initial expectations. Still, this combination even led to a decrease in the number of adventitious roots compared to data from explants cultured on medium with TIBA alone (Figure 2B). While the number of shoots was statistically significantly affected by the IAA at a concentration of 1 µmol L^−1^, this effect was negative. The results of the IAA and TIBA combination showed that no concentration of this combination had a significant effect on changes in shoot number, compared to data from explants cultured on medium with TIBA alone (Figure 2A).

The results showed that applying IAA to explants of *P. tremula* in vitro culture did not have a promoting effect on rooting, as described in the literature [12]. Even when using the TIBA and IAA combination, the effect of this hormone was opposite. Therefore, the other auxin, indolyl-3-butyric acid (IBA), was used as an antagonist. In terms of root number, IBA had a significant positive effect, using concentrations of 1 or 3 µmol L^−1^, compared to the control explants (Figure 2D). The effect of IBA in combination with TIBA was as expected, acting as an antagonist on the number of roots. It reversed the negative effect of TIBA, as compared to data from explants cultured in medium with TIBA. The lowest concentration of IBA used, 1 μmol L^−1^, resulted in a statistically significant decrease in the number of adventitious roots compared to the control explants and explants cultured on medium with TIBA (Figure 2D). For the morphological aspects of shoots, IBA and the combination of IBA and TIBA treatments had no significant effect on the number of shoots, compared to data from the control explants and explants cultured on medium with TIBA (Figure 2C).

Previous studies have shown that auxin is strongly associated with cytokinin, with endogenous auxin levels and their polar transport during in vitro shoot organogenesis being regulated by cytokinin [29,30]. Therefore, based on the auxin–cytokinin interaction, further studies were conducted to explore the influence of cytokinins on root development in aspen in vitro cultures.

### 2.2. The Influence of Cytokinin, BAP, on Root Formation

**Figure 3:**
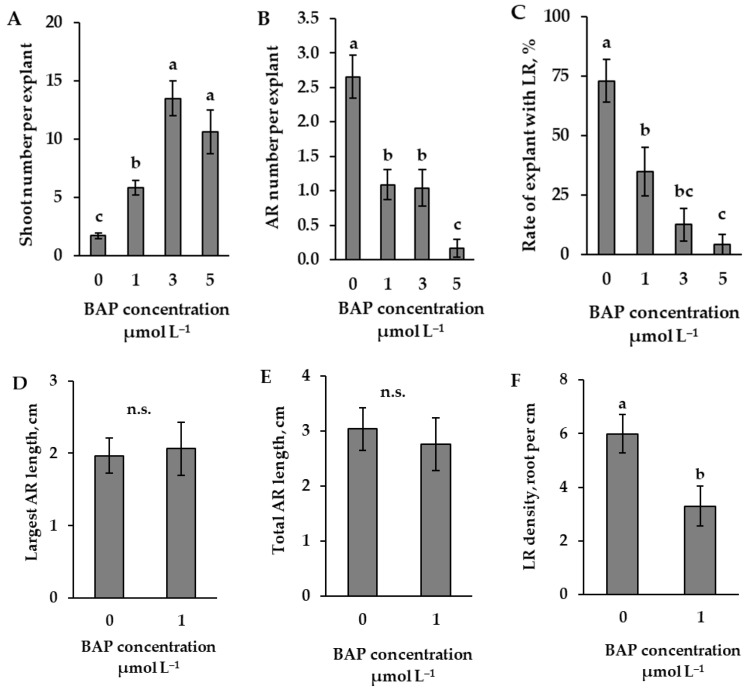
Shoots (**A**) and adventitious roots (AR) (**B**) per explant (means ± SE), the rate of explants with lateral roots (LR) (**C**), main (**D**) and total (**E**) adventitious root length (means ± SE) of explants, as affected by the presence of different BAP concentrations (0, 1, 3 and 5 µmol L^−1^) in the nutrient medium. (**A**–**C**) data are from the total number of explants or (**D**–**F**) from the number when the part of the explants with lateral roots exceeds ½ the total number. Lateral root density (LRD) (**F**) was calculated as the number of lateral roots per centimetre of cumulative adventitious root length (LRD = N/L). Significantly different means of samples grown under different nutrient media conditions are labelled with different letters (*p* < 0.05), n.s. indicates not significant (*p* > 0.05).

The results of the determination of optimal concentration of the phytohormone—cytokinin—6-benzylaminopurine (BAP)—as an inhibitor of root formation showed that one µmol L^−1^ was the lowest concentration that determined a statistically significant decrease in the number of adventitious roots in comparison with data from the control explants (Figure 3B). In terms of the number of shoots, all used concentrations of BAP resulted in a significant increase compared to the data from the control explants (Figure 3A). Analysis of the complete explant sample revealed that BAP significantly reduced the rate of explants with lateral roots. In this case, the concentrations of 1 and 3 µmol L^−1^ significantly decreased the rate of explants with lateral roots to 35% and 13%, respectively. In comparison, a concentration of five µmol L^−1^ decreased it to as low as 4% (Figure 3C). Accordingly, the data on the length of adventitious roots and the density of lateral roots (Figure 3D–F) were analysed using the lowest concentration of 1 µmol L^−1^ BAP. However, in this concentration effect, the rate of the sample with lateral roots did not exceed ½ of the total sample. The results from the sample of explants with lateral roots showed that one µmol L^−1^ BAP had no significant effect on the length of the largest root or the total length of adventitious roots in comparison with control explants (Figure 3D,E). Despite the unchanged length of adventitious roots, a concentration of 1 µmol L^−1^ BAP significantly reduced the density of lateral roots (Figure 3F). Considering the effect of BAP on root and shoot parameters, the most optimal tested concentration—1 µmol L^−1^—was selected for use in determining the optimal concentration of the IAA antagonist.

**Figure 4:**
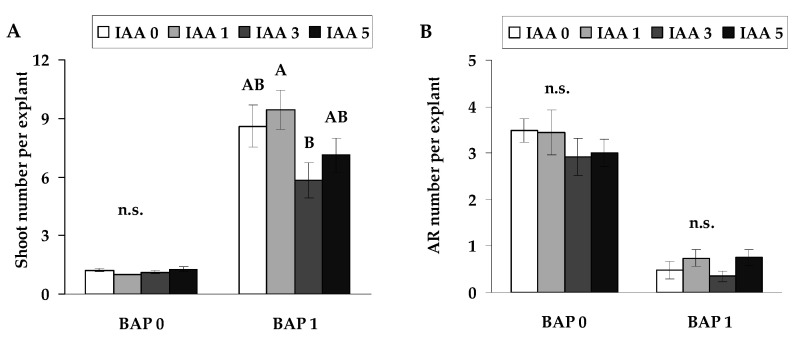
Shoots (**A**) and adventitious roots (AR) (**B**) per explant (means ± SE) of explants, as affected by the presence of IAA at the concentrations of 0, 1, 3 and 5 µmol L^−1^ combination without and with BAP (1 µmol L^−1^) in the nutrient medium. Significantly different means of samples grown under different nutrient media conditions are labelled with different letters (*p* < 0.05), n.s. indicates not significant (*p* > 0.05).

The application of IAA in combination with BAP did not result in significant changes in the number of adventitious roots compared to explants treated with BAP alone (Figure 4B). Although IAA is known to influence root development under certain conditions, in this experiment, it did not mitigate the inhibitory effect of BAP on rooting. Similarly, the combination of BAP and IAA at all tested concentrations had no significant effect on the number of shoots compared to explants grown on BAP alone (Figure 4A). These results indicate that IAA, at the tested concentrations, was not effective in modifying the response induced by BAP in either root or shoot formation.

### 2.3. The Influence of GA on Root Formation

**Figure 5:**
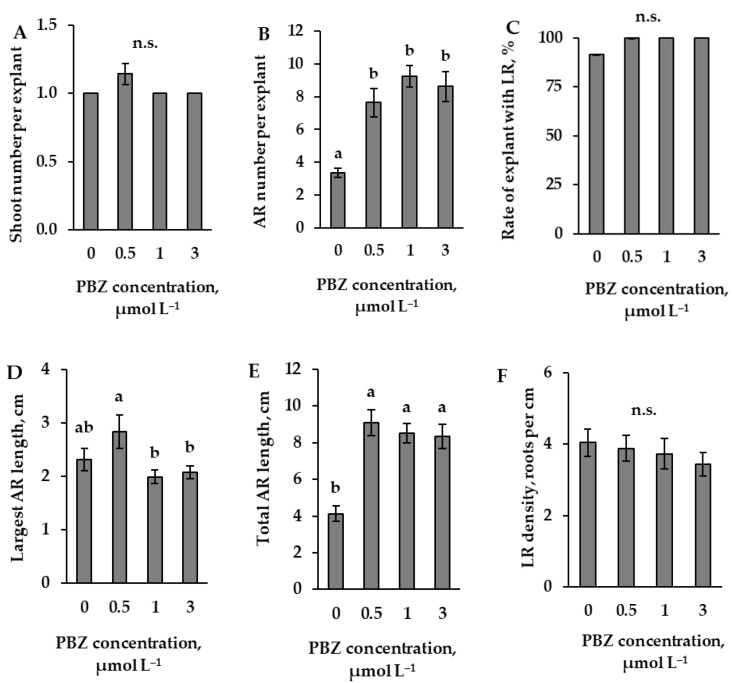
Number of shoots (**A**) and adventitious roots (AR) (**B**) per explant (means ± SE), the rate of explants with lateral roots (LR) (**C**), and the main (**D**) and total (**E**) adventitious root lengths (means ± SE) of explants, as affected by the presence of different PBZ concentrations (0, 0.5, 1 and 3 µmol L^−1^) in the nutrient medium. (**A**–**C**) data are from the total number of explants, or (**D**–**F**) from the number where the part of the explants with lateral roots exceeds ½ the total number. Lateral root density (LRD) (**F**) was calculated as the number of lateral roots per centimetre of cumulative adventitious root length (LRD = N/L). Significantly different means of samples grown under different nutrient media conditions are labelled with different letters (*p* < 0.05), n.s. indicates not significant (*p* > 0.05).

The results of the determination of the optimal concentration of paclobutrazol (PBZ), which inhibits the synthesis of gibberellin, showed that 0.5 μmol L^−1^ was the lowest of the used concentrations that resulted in a statistically significant change in the number of adventitious roots in comparison with data from the control explants (Figure 5B). All concentrations of PBZ used increased the number of adventitious roots; however, none of the concentrations used changed the number of shoots significantly (Figure 5A). The results of the data from the whole sample of explants indicate that all used concentrations of PBZ increased the percentage of explants with lateral roots to 100%. Therefore, the data for the length of the adventitious roots and the density of the lateral roots were analysed using all concentrations of PBZ, when the percentage of the sample with lateral roots was more than ½ of the total sample (Figure 5D–F). Sample data from the explants with lateral roots revealed that, in the case of length of the main adventitious root, none of the concentrations used, although significantly different from each other (from 0.5 to 1 and 3 μmol L^−1^), showed a significant effect in comparison with data from the control explants (Figure 5D). Although all concentrations of PBZ used increased not only the number of adventitious roots but also their total lengths (Figure 5E), none of the concentrations showed a significant effect on the density of the lateral roots (Figure 5F).

**Figure 6:**
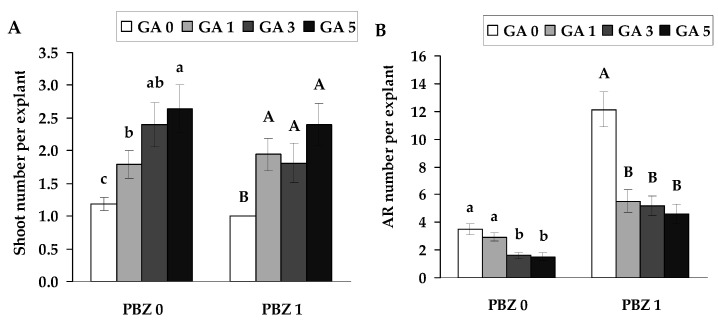
Shoots (**A**) and adventitious roots (AR) (**B**) per explant (means ± SE) of explants, as affected by the presence of GA_4+7_ at the concentrations of 0, 1, 3 and 5 µmol L^−1^ combination without and with PBZ (1 µmol L^−1^) in the nutrient medium. Significantly different means of samples grown under different nutrient media conditions are labelled with different letters (*p* < 0.05).

Given that PBZ inhibits gibberellic acid biosynthesis, the concentration of 1 μmol L^−1^ was selected to assess its influence on the physiological responses induced by GA_4+7_ application. The results showed that the positive effect of PBZ on root formation was significantly reduced when 3 or 5 μmol L^−1^ of GA_4+7_ was applied, indicating that GA_4+7_ acts as a functional antagonist to PBZ-induced stimulation of rooting (Figure 6B). Thus, when GA_4+7_ was used together with PBZ, it acted as an antagonist, significantly suppressing the PBZ-induced increase in root number compared to explants grown on medium supplemented with PBZ alone (Figure 6B). Regarding shoot number (Figure 6A), all concentrations of GA_4+7_ significantly increased shoot formation compared to control explants, suggesting that exogenous GA_4+7_ effectively promotes shoot development even under conditions where PBZ may inhibit gibberellin biosynthesis. When applied together with PBZ, GA_4+7_ significantly increased shoot number compared to explants cultured on PBZ alone, indicating that exogenous GA_4+7_ can overcome the inhibitory effect of PBZ on shoot development (Figure 6A).

## 3. Discussion

The mechanisms of adventitious root formation in *Populus* shoot in vitro culture have been under investigation for some time and continue to be studied to this day [7,31,32,33,34]. In our study, interpreting the obtained results was challenging, as although the mechanisms of adventitious root formation in *Populus* in vitro cultures have been explored for decades and remain a subject of ongoing research [18,31,32,33], specific comparative data remain limited. In the past decade, considerable attention has been devoted to investigating the genetic regulation of adventitious root formation [35,36,37,38,39]. Data on the investigation of these mechanisms using natural inhibitors of hormone transport or biosynthesis, as well as phytohormones that interfere with the signalling pathways of other phytohormones, remain limited. The results of this study regarding the key root formation phytohormone, auxin, were ambiguous, as the effects of the two types of auxins tested differed significantly. According to the previous studies on *P. tremula*, the auxin content of the whole plant strongly depends on the part of the shoot that contains the maximum amount, and auxin transport from the shoot to the root plays a key role in root formation [19,40,41]. Furthermore, explants from in vitro cultures of *Populus* mutants with larger leaves were found to develop a more robust adventitious root system [36]. In our study, the inhibition of auxin transport from shoots to roots by the application of TIBA is consistent with the results of previous research, as TIBA suppressed the formation and development of adventitious roots, an effect also observed in shoot tissues. The complexity of these mechanisms is highlighted by numerous genetic studies, including the identification of related genes and the investigation of *Populus* and other plant mutants [42,43]. Various other factors influencing auxin distribution in plants were also studied [27,44]. The complexity and significance of auxin transport is further demonstrated by our study, as the exogenous application of one of the auxins, IAA, unlike IBA, in the presence of TIBA does not replace the effect of endogenous auxin, which should have diffused from the shoot tissues, nor does it antagonise the negative effects of TIBA on root development. While most researchers report a positive influence of endogenous and exogenous IAA on root formation in *Populus*, our results stand out, as exogenously applied IAA did not affect root formation in *Populus* explants cultured in vitro [19,45]. This could be attributed to the characteristics of endogenous IAA concentration and the auxin transport mechanism, as well as its interactions with other bioactive molecules in the *Populus* explants studied; however, such claims require further investigation. The positive effect of IBA on the root system of hybrid aspen explants in vitro, as shown in Ref. [45], coincides with the results obtained in our study, where IBA positively influenced the development of adventitious roots in aspen. The results of our study suggest that the effects of different types of auxins on the development of *P. tremula* in vitro cultures should be distinguished based on whether endogenous or exogenous auxins modulate root development. Exogenous application of IAA and IBA did not significantly affect shoot development. However, when the transport of endogenously synthesised auxin from the shoot to the root was inhibited, leading to its accumulation in the shoot, enhanced shoot formation was observed. Although the effects of these two types of auxins differed, uncertainties remain regarding their role in root development. The neutral effect of exogenously applied auxin on shoot development, as well as the lack of effect of IAA on root formation, may be associated with its transport from the medium through the root to the shoot. In our experimental setup, we cultured the apical part of the plantlet, specifically a shoot segment with at least one bud and no pre-existing roots. During in vitro culture, roots developed de novo from the basal part of the explant. Our results indicate that the transport of endogenous auxin from these newly formed roots likely plays a crucial role in organ formation in the explant. Therefore, it could be argued that the transport of exogenous auxin from the medium, through the root, to the shoot may also have a decisive effect on its activity. This remains an open question for further research. In conclusion, the inhibition of auxin transport in aspen explants by TIBA cannot always be reversed by exogenous auxin application. Consistent with previous studies [45], our results suggest that, in in vitro aspen cultures, improved root system development can be achieved through the exogenous application of IBA or by promoting endogenous auxin synthesis and transport toward the root.

Studies on *Cedrela fissilis* Vell. have shown that TIBA, along with its interaction with other plant growth regulators, affects protein abundance, thereby influencing shoot and root development [34]. It has also been suggested that auxin transport can be modulated by all other phytohormones, proposing that this might be a primary function of phytohormones in plants. Studies show a strong association between auxin and cytokinin [29]. Other studies demonstrate that the endogenous auxin level and its polar transport during the organogenesis of in vitro shoots are dependent on cytokinin [30]. The root formation inhibitor, cytokinin, inhibited root development in aspen explants cultured in vitro, consistent with the results of other studies involving *Populus* shoots in vitro [12]. Although our results and those from previous studies demonstrated that cytokinins generally have a positive effect on shoot development, their efficiency can vary significantly depending on genotype, cytokinin type, and concentration. For example, some studies suggested that cytokinins may not always enhance shoot regeneration in certain *Populus* genotypes under in vitro conditions [46]. These data may be related to the negative effect of cytokinins on root formation, a phytohormone-regulated process, important for plant establishment in vitro. Although there is evidence that auxin suppresses cytokinin signalling in plants, in our study, the exogenous application of IAA auxin in the presence of BAP did not alleviate the negative effects of BAP on root development in aspen shoots cultured in vitro [47]. This could be attributed to the characteristics of endogenous IAA and BAP concentrations, as well as their interactions with other bioactive molecules in the studied *Populus* explants; however, such claims require further investigation. Therefore, considering the frequent use of cytokinins for shoot propagation in in vitro cultures, the external application of BAP in aspen cultures should be approached with caution and applied only when necessary, as it has been identified as a major phytohormone negatively affecting root system formation. The findings of this study confirm that exogenous application of IAA did not alleviate the inhibitory effects of BAP on root development. This aligns with the well-established understanding that BAP negatively impacts rooting parameters in aspen in vitro cultures. The combination of IAA and BAP did not enhance rooting, indicating that this interaction is insufficient to overcome the suppressive effects of BAP. While the use of IBA in combination with BAP has been suggested as a potential approach to improve rooting, this was not investigated in the present study and remains a subject for future research. Consequently, addressing the negative impact of BAP on root system formation remains a key challenge in optimising micropropagation protocols for aspen.

A key inhibitor of adventitious root formation, gibberellin, has a significant impact on explant development, as it not only inhibits root development but also affects shoot elongation. Our results are also consistent with those of other researchers, who have shown that gibberellins inhibit the formation of the *Populus* root system [27,35,48]. The promotion of *Populus* root formation by regulating gibberellin synthesis is investigated through transgenic mutants or by using the synthesis inhibitor paclobutrazol [27,35,49,50]. Our study results are also consistent with those of these researchers, as PBZ significantly enhanced root development in *Populus* shoots cultured in vitro. Notably, the exogenous application of PBZ in the presence of GA suppressed the negative effects of GA on aspen root systems. This demonstrates that PBZ has a highly significant impact on the aspen root system, directly linked to gibberellin.

## 4. Materials and Methods

### 4.1. Study Site and Experimental Material

For the study, shoot cultures of *P. tremula* clones, derived from a genotype selected in Lithuanian forests, were used as experimental material. The original donor tree was *P. tremula*, identified as code 18DPL037, listed in the 2015 database of the Lithuanian State Forest Service. The tree was located in a forest with the following geographic coordinates: 55°22′ N, 22°14′ E. In 2015, it was 60 years old, measuring 33 m in height and 0.64 m in diameter.

*P. tremula* shoots were initiated from twigs collected in early spring from the mid-crown of donor trees, before bud break. Shoot cultures were established following [31] and maintained in vitro through bimonthly subcultures on solidified Woody Plant Medium (WPM) [51] supplemented with 20 g L^−1^ sucrose and vitamins. During the first year, 0.5 mg L^−1^ 6-benzylaminopurine was included. Gelrite (4 g L^−1^) was used as the gelling agent. All media components were sourced from Duchefa Biochemie, Haarlem, The Netherlands.

### 4.2. Experiments on the Influence of Exogenously Applied PGR

All experiments were conducted using vitamin-supplemented WPM medium (Lloyd and McCown, 1980) [51], enriched with 20 g L^−1^ sucrose and solified with 4 g L^−1^ gelrite (Duchefa Biochemie, The Netherlands). Control groups were cultured on hormone-free medium, while experimental groups received specific plant growth regulators (PGRs): TIBA, IAA, IBA, BAP, PBZ, and GA_4+7_ (Duchefa Biochemie, The Netherlands). These plant growth regulators were used in the experiment at specified concentrations: TIBA at 1, 5, and 15 μmol L^−1^; IAA, IBA, and GA_4+7_ at 1, 3, and 5 μmol L^−1^; and PBZ at 0.5, 1, and 3 μmol L^−1^. The TIBA, IAA, and BAP powders were first dissolved in a drop of NaOH, IBA, and GA_4+7_ in ethanol, and PBZ was dissolved directly in distilled water. Each was then diluted with distilled water to an appropriate volume for the basal solution. The pH value of the basal solution of these chemicals was adjusted to 4.8 (the same as the medium). Solutions were sterile filtered (0.1 μm) and added to autoclaved medium (121 °C, 30 min; pH 4.8). Stem segments of approximately 2 cm in length were used as explants. Each explant contained either one apical node or two nodes (including one lateral node), ensuring at least one active meristematic region to support shoot regeneration. Single-stem explants were cultured vertically in glass tubes (150 × 20 mm) with 5 mL of medium, under a 16 h photoperiod (30 µE m^−2^ s^−1^) at 25 °C/18 °C (day/night). Tubes were placed in wooden pallets to reduce light exposure to the medium base (~3 cm), minimising photodegradation of compounds. Figure S1 presents examples of experiments illustrating shoot and root development (A–D), the regeneration room (E), and the effects of inhibiting auxin transport on root development (F).

Each explant was treated and measured as an individual biological replicate. For each treatment, 30 explants were included in a single experimental run, and their morphological parameters were analyzed independently to assess treatment effects. Cultures were evaluated after 2 months. For each explant, the number of shoots, adventitious roots, and lateral roots was recorded visually. Adventitious root lengths were measured with a ruler. Lateral root density (LRD) was calculated as the number of lateral roots (N) per centimetre (L) of the cumulative length of adventitious roots in cm, using the formula LRD = N/L. Data analysis was based on chemical concentration and focused on the proportion of explants forming lateral roots exceeding half of the total root system, expressed as a percentage.

### 4.3. Experimental Design and Data Analysis

The influence of exogenously applied hormone transport or biosynthesis inhibitors, as well as antagonistic hormone combinations, on shoot and adventitious root formation in *P. tremula* (genotype 18DPL037) was investigated. Morphological root parameters were evaluated to determine whether specific phytohormones or inhibitors had a statistically significant effect, indicating their regulatory role in root system development. In each experiment, optimal concentrations of individual plant growth regulators (PGRs) and their antagonists were identified. The optimal PGR concentration was defined as the lowest concentration inducing a statistically significant increase or decrease in adventitious root number compared to the control. The optimal antagonist concentration was the lowest that, when combined with the PGR, restored root numbers to a level not significantly different from control. Tested combinations included TIBA with IAA or IBA; BAP with IAA; and PBZ with GA_4/7_. Experimental groups consisted of (1) control (hormone-free medium), (2) PGR only, (3) PGR + antagonist, and (4) antagonist only.

### 4.4. Statistics

Comparative analysis was performed using a two-tailed Welch’s *t*-test, appropriate for samples with unequal variances [52], using Microsoft Excel 2010. Rates (e.g., proportion of explants exhibiting a given trait) were treated as binomial distribution means. Differences between groups were considered statistically significant at *p* < 0.05.

## 5. Conclusions

The results showed that the optimal concentrations of antagonists did not fully restore the effects of the optimal concentration of plant growth regulators. It was determined that the optimal concentrations of plant growth regulators that determine the formation of adventitious roots of aspen (*P. tremula*) in vitro culture were 2,3,5-triiodobenzoic acid (TIBA)—5 µmol L^−1^, benzyl aminopurine (BAP)—1 µmol L^−1^, and paclobutrazol (PBZ)—1 µmol L^−1^. The formation of adventitious roots in *P. tremula* can be specifically inhibited by supplementing the medium with auxin transport inhibitor 2,3,5-triiodobenzoic acid. It can be promoted by using an inhibitor of gibberellin synthesis, paclobutrazol.

For optimal root system development in in vitro aspen cultures, it is advisable to apply exogenous IBA or enhance endogenous auxin synthesis and its transport to the roots. Given the frequent use of cytokinins for shoot propagation, caution is recommended when applying BAP, as it can significantly hinder root formation.

## Data Availability

The original contributions presented in this study are included in the article. Further inquiries can be directed to the corresponding author.

## Supplementary Materials

The following supporting information can be downloaded at: https://www.mdpi.com/article/10.3390/plants14152427/s1, Figure S1: Photos show examples of experiments on shoot and root development (A–D), the regeneration room (E), and the effects of auxin transport on root growth (F). Photos by Miglė Vaičiukynė.

## Abbreviations

The following abbreviations are used in this manuscript:

AR	Adventitious roots
BAP	6-Benzylaminopurine
GA_4+7_	Gibberellins A_4_ and A_7_
IAA	Indole-3-acetic acid
IBA	Indole-3-butyric acid
LR	Lateral roots
PBZ	Paclobutrazol
TIBA	2,3,5-Triiodobenzoic acid
WPM	Woody Plant Medium
