# Supplementary material for: Role of Plant Growth Regulators in Adventitious Populus Tremula Root Development In Vitro
Source: Plants (Basel). 2025 Aug 5;14(15):2427. doi: 10.3390/plants14152427 (PMC12349478; doi:10.3390/plants14152427)
Supplement: Supplementary file 1 [file plants-14-02427-s001.zip › plants-3765050-supplementary.pdf]

plants

Figure S1. Photos show examples of experiments on shoot and root development (A–D), the regeneration room (E), and the effects of auxin transport on root growth (F). Photos by Miglė Vaičiukynė.

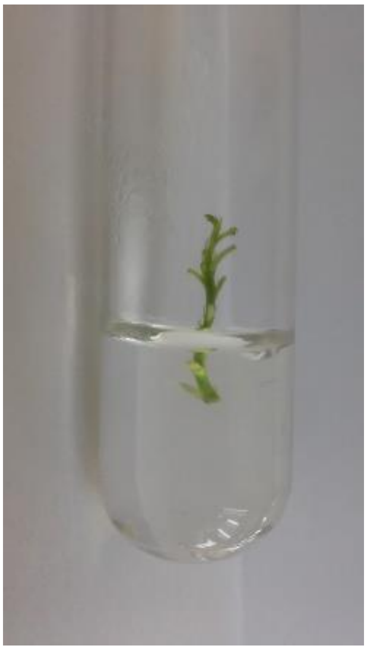

A – *Populus tremula* explant

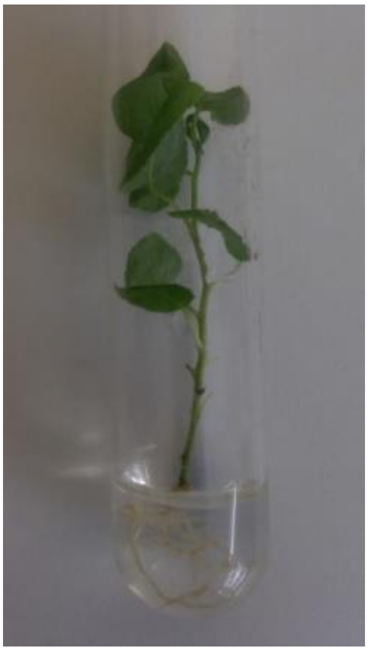

B – *Populus tremula* explant with one shoot

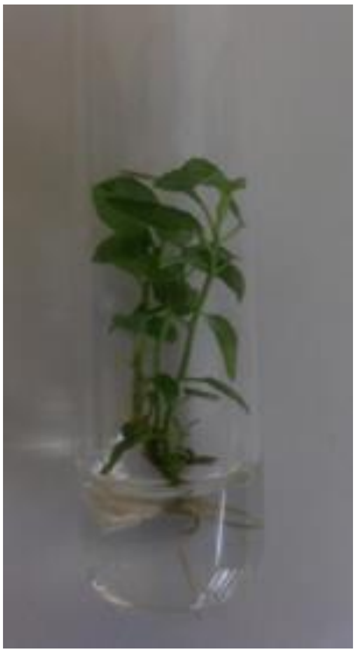

C – *Populus tremula* explant with two shoots

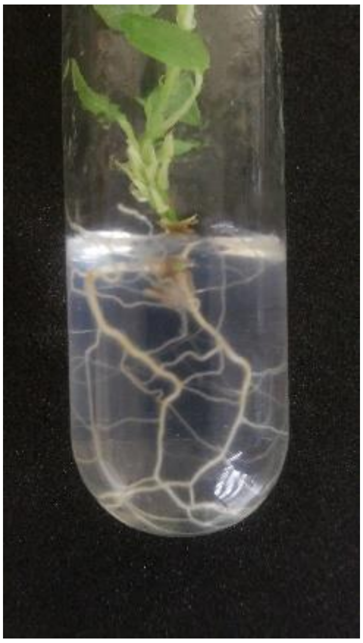

D – *Populus tremula* roots

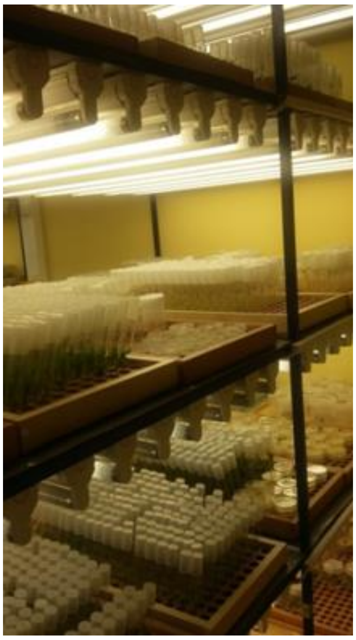

E – Regeneration room

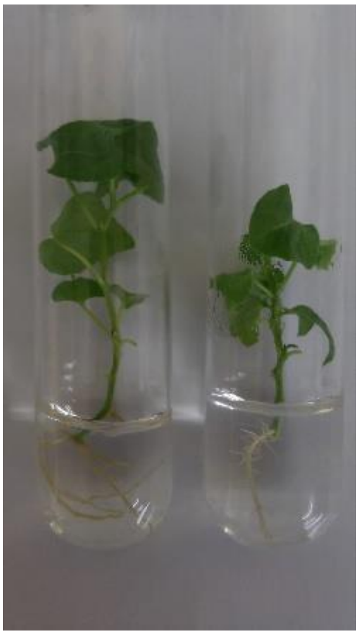

F – Effect of auxin transport inhibition on root formation: explants from the Control group (left) and from medium supplemented with 15 µmol L^{-1} TIBA (right).
